# Genome differentiation in a species pair of coregonine fishes: an extremely rapid speciation driven by stress-activated retrotransposons mediating extensive ribosomal DNA multiplications

**DOI:** 10.1186/1471-2148-13-42

**Published:** 2013-02-14

**Authors:** Radka Symonová, Zuzana Majtánová, Alexandr Sember, Georg BO Staaks, Jörg Bohlen, Jörg Freyhof, Marie Rábová, Petr Ráb

**Affiliations:** 1 Laboratory of Fish Genetics, Institute of Animal Physiology and Genetics, Czech Academy of Sciences, Rumburská 89, 277 21, Liběchov, Czech Republic; 2 Leibniz-Institut of Freshwater Biology and Inland Fisheries, Müggelseedamm 310, 12587, Berlin, Germany

## Abstract

**Background:**

Sympatric species pairs are particularly common in freshwater fishes associated with postglacial lakes in northern temperate environments. The nature of divergences between co-occurring sympatric species, factors contributing to reproductive isolation and modes of genome evolution is a much debated topic in evolutionary biology addressed by various experimental tools. To the best of our knowledge, nobody approached this field using molecular cytogenetics. We examined chromosomes and genomes of one postglacial species pair, sympatric European winter-spawning *Coregonus albula* and the local endemic dwarf-sized spring-spawning *C*. *fontanae*, both originating in Lake Stechlin. We have employed molecular cytogenetic tools to identify the genomic differences between the two species of the sympatric pair on the sub-chromosomal level of resolution.

**Results:**

Fluorescence *in situ* hybridization (FISH) experiments consistently revealed a distinct variation in the copy number of loci of the major ribosomal DNA (the 45S unit) between *C*. *albula* and *C*. *fontanae* genomes. In *C*. *fontanae*, up to 40 chromosomes were identified to bear a part of the major ribosomal DNA, while in *C*. *albula* only 8–10 chromosomes possessed these genes. To determine mechanisms how such extensive genome alternation might have arisen, a PCR screening for retrotransposons from genomic DNA of both species was performed. The amplified retrotransposon *Rex1* was used as a probe for FISH mapping onto chromosomes of both species. These experiments showed a clear co-localization of the ribosomal DNA and the retrotransposon *Rex1* in a pericentromeric region of one or two acrocentric chromosomes in both species.

**Conclusion:**

We demonstrated genomic consequences of a rapid ecological speciation on the level undetectable by neither sequence nor karyotype analysis. We provide indirect evidence that ribosomal DNA probably utilized the spreading mechanism of retrotransposons subsequently affecting recombination rates in both genomes, thus, leading to a rapid genome divergence. We attribute these extensive genome re-arrangements associated with speciation event to stress-induced retrotransposons (re)activation. Such causal interplay between genome differentiation, retrotransposons (re)activation and environmental conditions may become a topic to be explored in a broader genomic context in future evolutionary studies.

## Background

Intra-lacustrine fish speciation as an example of ecological speciation is a much debated topic in evolutionary biology addressed by various experimental tools, mostly in complex systems with a number of species, in particular in ancient freshwater lakes [1]. In Europe, with its comparatively depauperate fish fauna, issues of adaptive radiation and ecological speciation in fishes are highly relevant in temperate postglacial lakes (originating after the last glaciation i.e. 12–15 kyrs BP). To assess potential modes of speciation in fishes, numerous model systems are available [2], among which one of the best groups with a robust knowledge on adaptive speciation and complex speciation patterns in postglacial lakes are coregonine fishes (Coregoninae, [3]) [4-6]. Within coregonines, their numerous sympatric species pairs and recent species flocks [7-9] are of particular importance [10]. In *Coregonus*, based on extensive genetic and population genetic [11], phylogenetic, biogeographic, morphological and eco-physiological data, six potential modes of speciation have been proposed [12]. However, none of these approaches utilized cytogenetic data despite salmonid fishes, to which coregonines belong, being one of the best karyologically studied fish groups in terms of the number of species, populations, individuals and material (adults and embryos) examined. Available cytogenetic data demonstrate that salmonids include two basic karyotypes – the high chromosome number 2n ~ 80 (type A and its derivatives) and the low chromosome number 2n ~ 60 (type B and its derivatives) – co-occurring in all recognized salmonid phylogenetic lineages (except graylings, Thymallinae), including whitefish, ciscoes and innconu (Coregoninae). Species with the type B karyotypes have in common either prominent anadromous behaviour and/or are found in lacustrine environments and are likely products of intra-lacustrine speciation (for review [13]). Such apparent parallelism might be explained by specific life history strategies leading in both types of environments to small effective population sizes, thus, enabling increased probability of fixation of genic or chromosomal mutations. Observed evolution of chromosome number in salmonids is likely affected by selection for increased or decreased genetic recombination rate as proposed by Quimseyh [14], explaining high variability in chromosome numbers in mammals based on fundamental numbers (NF, chromosome arms number).

In this study, we examined chromosomes and genomes of the sympatric species *Coregonus albula* and *C*. *fontanae* in the dimictic Lake Stechlin, northern Germany to test whether the above outlined parallelism on karyotype differentiation in intralacustrine species pairs can also be observed in incipient speciation processes in young postglacial lakes. Both species are pelagic zooplanktivores, but they differ considerably in their size, spawning time [15] and temperature-dependent metabolic physiological adaptations [16]. Up to now, *C*. *fontanae* has not yet been subjected to any cytogenetic analysis as opposed to *C*. *albula* (see [17] and references therein). The level of genetic differentiation between *C*. *albula* and *C*. *fontanea* tested by combined analyses of mitochondrial DNA and microsatellite loci showed a weak differentiation (*F*_ST_ = 0–0.008) between these two species when compared with another sympatric species pair *C*. *albula* and *C*. *lucinensis*[18]. Further population genetic analyses based on 1244 polymorphic AFLP loci demonstrated a lower differentiation between allopatric than sympatric populations of the *C*. *albula* complex and suggested a rather complex colonization history than simple sympatric speciation [6]. Therefore, we have employed a novel approach in this field to explore the up to now neglected aspects of genome evolution in this species pair and used different parts of ribosomal DNA of the 45S rDNA unit as cytotaxonomic markers.

**Figure 1:**
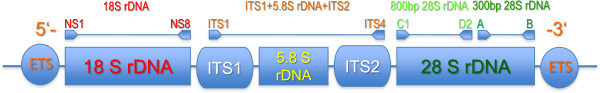
**Schematic representation of the 45S rDNA unit and FISH probes construction including primers nesting sites.** (not to scale).

At the first stage of this study, we have employed conventional methods of karyotype analysis (Giemsa and Ag staining, CMA_3_ and DAPI fluorescence). At the second stage, we have performed molecular cytogenetic analyses (CGH and FISH with various rDNA fragments and non-LTR retrotransposons as probes) to identify any differences between chromosomal complements of these two species on the sub-chromosomal level of resolution since the karyotype analyses showed no significant differences. At the third stage, we performed molecular biological analyses of the 45S ribosomal RNA genes and the *Rex1* non-LTR retrotransposon. Furthermore, we discuss these results in the context of populations of small effective sizes under extreme stress conditions under which retrotransposons (re)activation could have contributed to accelerated speciation.The major cluster of ribosomal RNA genes is expressed as the 45S transcriptional unit (Figure 1). This unit consists of 18S, 5.8S and 28S rDNA genes, separated by internal transcribed spacers (ITS1, ITS2) and surrounded by external transcribed spacers (ETS). The 45S rDNA units are arranged in tandem repetitions with high copy numbers [19,20] therefore, they represent a useful cytotaxonomic marker. The individual units are separated by intergenic spacers (IGS) [21,22]. The structure and the order of genes within the unit are highly conserved among Eukaryota [23]. Different parts of the 45S transcriptional unit display different mutational rates. The most conserved region is the 18S rRNA gene and the most variable are ITSs [23].

## Results

### Karyotyping and comparative cytogenetics

**Figure 2:**
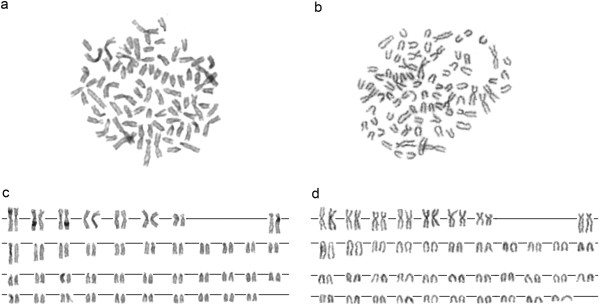
**Giemsa-stained metaphase plates and corresponding karyogram of *C. albula *(a, c) and *C. fontanae *(b, d).** Bar = 5 μm.

**Figure 3:**
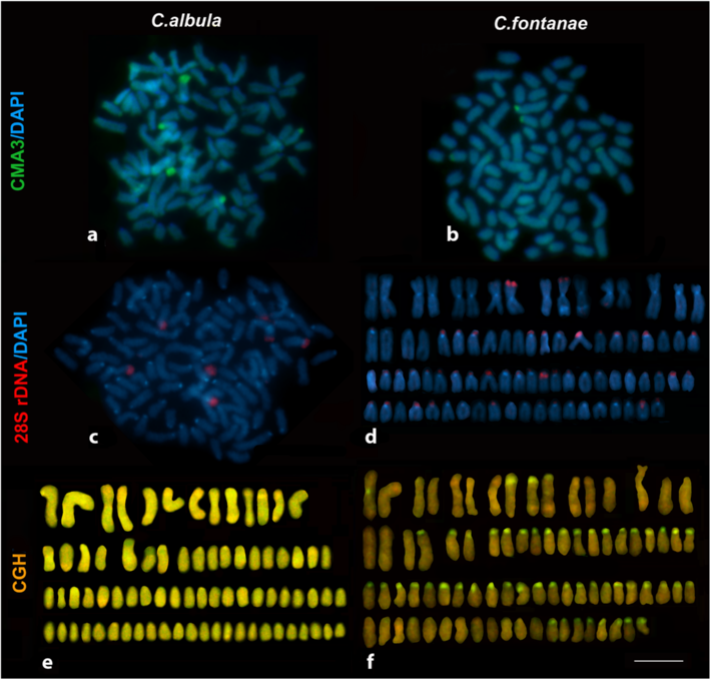
**Metaphase plates and karyograms of *C. albula *and *C. fontanae *showing Chromomycin A**_**3**_**/DAPI staining, FISH and CGH experiments.** Chromomycin A_3_ (CMA_3_) fluorescent staining (green) and DAPI (blue) staining in *C. albula* (**a**) and *C. fontanae* (**b**). FISH with the 28S rDNA (300 bp probe) (red), DAPI counterstaining (blue) in *C. albula* (**c**). FISH with the 28S rDNA (800 bp probe) (red), DAPI counterstaining (blue) in *C. fontanae* (**d**). A set of reciprocal comparative genomic hybridization (CGH) experiments to *C. albula* chromosomes (**e**) and *C. fontanae* chromosomes (**f**). In both (**e**, **f**), the *C. albula* genomic DNA was labelled in red and the *C. fontanae* genomic DNA in green. Bar = 5 μm.

Karyotypes of both examined ciscoes were very similar (2n = 80 in both species) and both belong to the karyotype category A *sensu*[13]. They both had 8 pairs of meta- (m) to submetacentric (sm) and 32 pairs of acrocentric (a) chromosomes (both sexes in *C*. *fontanae*, only males in *C*. *albula* were available), The NF was 96 in both species (Figure 2a-d). The sequential Chromomycin A_3_ (CMA_3_, particularly specific for CG rich regions) and DAPI (specific for AT rich regions) stainings revealed in both species a varying number of 6–8 sites with CMA3^+^/DAPI^-^ signals. The signals occurred at telomeric regions of 3–4 metacentric chromosomes and at pericentromeric regions of 3–4 acrocentric/submetacentric chromosomes (Figure 3a, b). In some nuclei, several other weakly CMA_3_^+^ regions not corresponding to DAPI^-^ signals mostly with pericentromeric locations were observed (Figure 3a). This variability occurs on the inter-individual as well as on the intra-individual level.

### Cytogenetic mapping of ribosomal DNA and comparative genomic hybridization (CGH)

**Figure 4:**
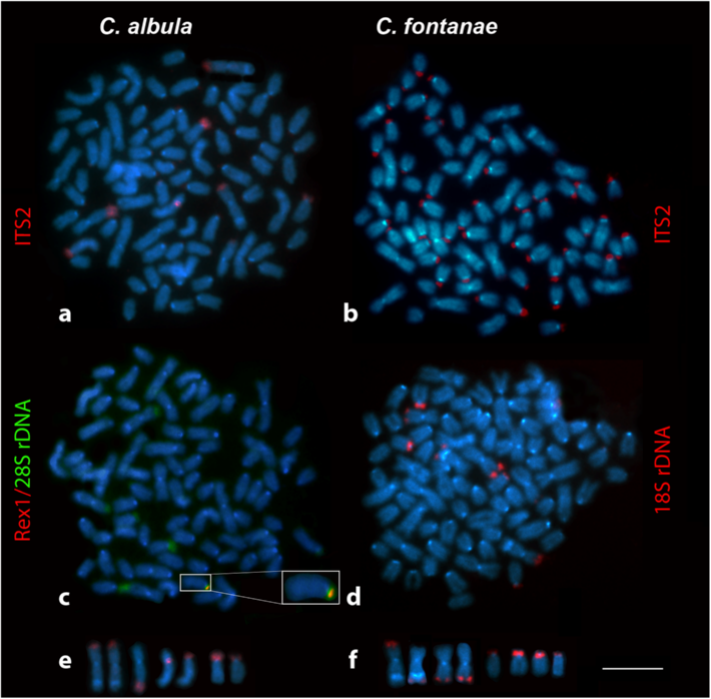
**Metaphase plates and selected chromosomes of *C. albula *and *C. fontanae *showing FISH experiments.** FISH with ITS2 (red) as probe hybridized to *C. albula* (**a**) and to *C. fontanae* (**b**), counterstained with DAPI. Double-FISH analysis with the *Rex1* retrotransposon (red) and the 800 bp 28S rDNA (green) to *C. albula* (**c**), detail of the chromosome with *Rex1* and 28S rDNA co-localization in inset. FISH with the 18S rDNA (red) to *C. fontanae* chromosomes (**d**) counterstained with DAPI. Chromosomes bearing the ITS2 (red) signal in *C. albula* (**e**). Chromosomes bearing the 18S rDNA signals (red) in *C. fontanae* (**f**). Chromosomes on (**f**) represent the hypothetically ancestral condition of rDNA distribution prior to its multiplication in *C. fontanae*. Bar = 5 μm.

Fluorescence *in situ* hybridization (FISH) with 28S ribosomal DNA (rDNA) probes derived from two non-overlapping regions of the 28S rRNA gene of both species (an 800 bp region adjacent towards the 5′-end of the 28S rDNA gene and a 300 bp region adjacent towards the 3′-end) showed strikingly different results. FISH using the shorter fragment as a probe revealed the presence of 6–10 chromosomes in both *C*. *albula* and *C*. *fontanae* bearing such sequences distributed similarly as the CMA_3_^-^/DAPI^-^ (Figure 3c for *C*. *albula* only). FISH with the longer fragment revealed bright signals on 6–10 chromosomes in *C*. *albula* (shown in co-localization with *Rex1* retrotransposon, Figure 4c) but up to 40 signals (varying numbers) on chromosomes in *C*. *fontanae* (Figure 3d). Most of the signals of the 800 bp probe of the 28S rDNA in *C*. *fontanae* were localized in the AT rich (i.e. DAPI^+^) centromeric or pericentromeric regions of acrocentric chromosomes. Two signals of the 800 bp 28S rDNA probe corresponded to the major NOR sites evidenced also by the 300 bp rDNA and the CMA_3_/DAPI staining that were localized in telomeric regions of two large metacentric chromosomes (Figure 3d).

To verify these striking differences between *C*. *albula* and *C*. *fontanae*, we carried out a set of reciprocal comparative genomic hybridization (CGH) experiments. A mixture of the whole genome DNA (gDNA) of both *C*. *albula* and *C*. *fontanae* was hybridized simultaneously to both *C*. *fontanae* and *C*. *albula* chromosomes. This resulted in nearly no significant differences on *C*. *albula* chromosomes, i.e. a balanced hybridization of both gDNA probes was observed (Figure 3e). While signal of the *C*. *fontanae* gDNA when *in situ* compared with the *C*. *albula* gDNA onto *C*. *fontanae* chromosomes was distinctly overrepresented in mostly pericentromeric regions of about 40 chromosomes (green signals in Figure 3f). This pattern corresponded to results of the FISH experiment with 800 bp 28S rDNA to *C*. *fontanae* chromosomes.

To assess quantitative differences in the distribution of the whole 45S rDNA unit in both species, a further set of comparative FISH experiments with a cocktail of the 18S rDNA and ITS1-ITS2 (including 5.8S rDNA) as probes amplified from both of the genomes were performed to *C*. *albula* and *C*. *fontanae* chromosomes. In the genome of *C*. *albula*, both the ITS1 and ITS2 were present in 6–12 signals with a varying number of signals (Figure 4a). In the genome of *C*. *fontanae*, both the ITS1 and ITS2 were multiplied to the same extent as the 800 bp 28S rDNA part, i.e. a varying number of approximately 40 signals (Figure 4b). The subsequent FISH experiment with the 18S rDNA in both species showed the number of 6–10 signals (Figure 4d for *C*. *fontanae* only). The typical chromosomes bearing rDNA signals in the unamplified condition (i.e. ITS1-ITS2 and 28S rDNA in *C*. *albula* and 18S rDNA in *C*. *fontanae*) are shown in Figures 4e-f. There is a reproducible difference in location of one of the 28S rDNA in *C*. *fontanae* (when compared with *C*. *albula*) related to a distinct DAPI^+^ band on a large metacentric chromosome pair (Figure 4f). In *C*. *albula*, the rDNA signal was always located on the opposite arm than the DAPI^+^ band occurred (Figure 4e). In *C*. *fontanae*, one signal is located on the same arm and one signal is on the opposite one (Figure 4f). The construction of the FISH probes used in this study is visualized in Figure 1.

### Molecular characterization of multiplied rDNA sites

To determine mechanisms how such extensive multiplication of parts of rRNA genes in *C*. *fontanae* might have arisen, a PCR screening for non-LTR retrotransposons in genomic DNA of both species was performed. Retrotransposons of the *Rex* family are known to have invaded fish genomes in multiple lineages [24] and to also insert into rDNA, particularly in fishes [25]. Therefore, the retroelements *Rex1*, *Rex3* and *Rex6* were tested in this study. FISH with the *Rex3* and *Rex6* retroelements yielded inconclusive results. The *Rex1* element, as a probe hybridized to chromosomes of *C*. *albula* and *C*. *fontanae*, typically showed a dispersed pattern of signals on all chromosomes with a distinct accumulation in a pericentromeric region of one single acrocentric chromosome. Co-hybridization of the *Rex1* element with the 800 bp 28S rDNA probe in a double-FISH experiment showed co-localization of these two probes typically on one (exceptionally two to several), mostly acrocentric chromosomes in both *C*. *albula* and *C*. *fontanae* (Figure 4c for *C*. *albula* only, detail of the co-localization in inset). The *Rex1* signal with a distinctly weaker intensity occurred dispersed also on other sites corresponding to the NOR loci in both genomes. The sequences of the *Rex1* derived from the *C*. *albula* and *C*. *fontanae* genome were deposited in GenBank under the accession numbers JQ731754 and JQ731760, respectively.

Sequencing of the 18S and 28S rDNA, as well as ITS1 and ITS2 (deposited in GenBank under accession numbers JQ731749-JQ731753 and JQ731755-JQ731759) yielded no significant differences in these genes between *C*. *albula* and *C*. *fontanae*.

## Discussion

Our findings of extensive genomic re-arrangements of a substantial fraction of the 45S rDNA unit in the *C*. *fontanae* genome when compared with the situation in *C*. *albula* are in strong contrast with previously reported low genetic differentiation between these two species [6,18].

Our results indicate that in the genome of *C*. *fontanae* next to the complete 6–10 NOR loci corresponding to similar number of NOR-bearing chromosomes in *C*. *albula*, up to 30 supernumerary and incomplete NOR loci occur. This is supported by results of the sequential fluorescent staining (CMA_3_ and DAPI), showing about 6–8 signals in karyotypes of both species, although on chromosomes of *C*. *fontanae* the signals were slightly weaker. However, these supernumerary sites in *C*. *fontanae* were not represented by repeating of the complete 45S rDNA unit (i.e. 18S rDNA, ITS1, 5.8S rDNA, ITS2, 28S rDNA)_n_ but only by a part including probably complete ITS1 and ITS2 and a part of the 28S rDNA adjacent to the ITS2, i.e. the 5′ end of the 28S rDNA gene (the region of 5.8S rDNA was not investigated separately).

Most of the supernumerary signals of the 45S rDNA in chromosomes of *C*. *fontanae* were localized in the AT rich pericentromeric regions as well as the major accumulation of the *Rex1* retrotransposon on both *C*. *albula* and *C*. *fontanae* chromosomes. This is in accordance with findings of other authors describing accumulations of transposable elements in centromeric heterochromatin e.g. in genome of humans [26] and in a cichlid fish *Cichla kelberi*[27,28]. TEs in fishes generally tend to insert to heterochromatic areas of chromosomes ([29]; reviewed by [30]). There are also records of specific integration of some non-LTR retrotransposons at the rRNA genes found in most animal phyla (summarized by [31]), in insects *Drosophila melanogaster* and *Bombyx mori*[32] or in the fish *Erythrinus erythrinus*, where *Rex3* retrotransposons were found in the 5S rRNA genes [25].

In the above-mentioned *E*. *erythrinus* fish, a similar multiplication of rRNA genes was described [25]. In that case, four karyomorphs of *E*. *erythrinus* differ in their chromosomal number, karyotype, presence or absence of heteromorphic sex chromosomes and numbers of 5S rDNA loci. The karyomorph A in *E*. *erythrinus* showed only two 5S rDNA loci, while in the karyomorph D, 21–22 5S rDNA loci could be observed. All 5S rDNA sites co-localized with the *Rex3* retrotransposon. On the other hand, no changes in the heterochromatin and 18S rDNA patterns were found between these two karyomorphs [25]. Such two karyomorphs within a single species *E*. *erythrinus* may be seen as an incipient stage of a speciation event. This situation can thus represent an initial stage, later resulting in the condition observed in morphologically [15], ecologically and physiologically [33,34] diverged species pair *C*. *fontanae* and *C*. *albula* described in this study. A similar observation of extremely multiplied NOR sites (46 and 49 countable FISH signals), however, without any further detailed analysis, were reported in brook char *Salvelinus fontinalis* (Salmonidae) [35].

In salmonid fishes, TEs have been studied intensively [30,36,37]. Microarray studies showed that transcription of rainbow trout transposons is activated by external stimuli, such as toxicity, stress and bacterial antigens [38]. In the oligotrophic Lake Stechlin, the food availability for coregonines was extremely limited and the size at maturity and the maximal size of *C*. *albula* are far behind the other populations of this species in adjacent lakes in northern Germany [39]. *C*. *fontanae* is the smallest species of the genus *Coregonus* in Europe [8]. Raising both species in the laboratory demonstrated that both grew much larger if supported with unlimited food (unpublished obs., Freyhof). Therefore, it can be speculated that both species, especially *C*. *fontanae*, live in an extreme permanent starvation in the Lake Stechlin. It can be also hypothesized that the spring-spawning habit of *C*. *fontanae* might have originated simply by the shift of sexual maturity in the part of the population that has not been able to attain sexual maturity in autumn due to the lower food intake and hence environmental starvation stress.

### Link between environmental stress and chromatin modification/regulation

Effects of stress on the genome can result in important perturbations creating new combinations better compatible with survival (summarized by [40]; more recently reviewed by [41]). After the discovery of transposable elements (TE) more than 50 years ago, their mutagenic effect had been increasingly viewed in association with rapid genome reorganizations by the creation of new regulation patterns and chromosome restructuring during last years [41]. Stress activated mobilization of these elements by failure of epigenetic silencing (the host defence model of repressing the movement of mobile elements; [42,43]) can lead to (re)activation of mobile elements and consequently to major and rapid genome alterations [40,41,44,45].

Barbara McClintock [46] already considered TE as a source of hypermutagenicity creating viable and fertile individuals from a stressed population under risk of extinction. Moreover, she originally named TE “controlling elements” due to their ability to alter gene activity and genome structure [47].

### TE-mediated genome rearrangements as a factor in speciation

With growing evidence for the importance of TEs in the genome evolution, the role of TE-mediated genome changes in the speciation by their possible contribution to pre- and post-mating reproductive isolation formation has been increasingly taken into account and discussed generally in eukaryotes [48,49], *Drosophila*[50], fishes [51], mammals [52], and plants [53]. However, lack of experimental data makes it difficult to prove this possibility (reviewed by [51,54,55]). On the other hand, [41] provides an overview of TE transposition bursts concomitant with radiation periods in seven cases. The same authors also discuss TE-induced rapid speciation associated with the ability of TEs to induce chromosomal rearrangements. Therefore, the sympatric species pair *C*. *albula* and *C*. *fontanae* in the context of other congeneric coregonine species and their variable evolutionary history in the Eurasian post-glacial lakes appears to be a suitable model system for exploring mechanisms of genomic differentiation and speciation with or without TE contribution.

In a very similar, but North American study system (lake whitefish species pairs, *Coregonus* spp.), [56] next generation sequencing (NGS) showed that TEs appeared to be highly expressed in hybrids between two recently diverged species. This may be potentially the mechanism responsible for post-zygotic reproductive isolation. Moreover, NGS can be viewed as a useful tool complementary with molecular cytogenetic approach presented in this study enabling confirmation of here documented results and search for other candidate groups of TEs involved in the genome re-arrangements and accelerated speciation.

## Conclusion

In the sympatric species pair *C*. *albula* and *C*. *fontanae*, we encounter a complex situation involving several evolutionary phenomena and factors. Firstly, a rapid ecological speciation event with an unclear sympatric scenario, i.e. the derived species *C*. *fontanae* fully differentiated from *C*. *albula* physiologically, ecologically and morphologically within about 12 – 14 kyrs in the newly colonized Stechlin Lake after the last glacier retreated [15]. Secondly, genetic differentiation of these two species remained weak as the combined analyses of mtDNA and microsatellite loci [18] showed, as well as major karyotypic and chromosomal markers presented in this study. This is in contrast with extensive genome re-arrangements in a large proportion of the 45S rDNA cassette in *C*. *fontanae* when compared with its most likely ancestral species – *C*. *albula*. The genome re-arrangements are exhibited as a distinct loci number differences and relocation of variable number (about 30) AT rich pericentromeric regions in *C*. *fontanae*. The molecular mechanism behind these re-arrangements might be a retrotransposition of a part of the 45S rDNA unit mediated by retrotransposons. Retrotransposonal activity can be mobilized under certain conditions (stress, environmental changes) and cause rapid and extensive structural changes to the host genome. These structural genomic differences in *C*. *fontanae* accumulated to pericentromeric heterochromatin in almost half of the chromosome complement. This might then have been acting as a partial but permanent reproductive barrier by hampering recombination, thus, enabling and accelerating the morphological, ecological and physiological differentiation of *C*. *fontanae*. Moreover, interspecific hybridization between the old and the newly arising species might have activated retrotransposonal activity in hybrids resulting in hybrid sterility or unviability as reviewed by [51]. The population genetic parameters of this speciation event, favouring fixation of the re-arranged genomes, remain to be elucidated in detail, but small effective population size is a good hypothesis to be tested.

## Methods

### Materials

For this study, we had 12 individuals of *Coregonus albula* (Linnaeus, 1758) and 16 individuals of *C*. *fontanae*[15], both from Lake Stechlin (northern Germany, Brandenburg, 53° 10’ N; 13° 02’ E). All fish were raised in the laboratory under identical conditions as described by [33,34]. In *C*. *albula*, 3 individuals (samples alb 1, 2 and 5, males only) yielded metaphases usable for down-stream FISH and CGH experiments. In *C*. *fontanae*, 3 individuals also (samples font 2, 5 and 7, both males and females) yielded usable chromosome preparations. Of all studied individuals, we isolated genomic DNA from fin clips and muscles. All tissue and DNA samples, including cell suspensions and chromosome preparations, are deposited in the Laboratory of Fish Genetics of the Institute of Animal Physiology and Genetics (IAPG). This study was covered by the “Valid Animal Use Protocols” Nr. CZ 00221 at the IAPG issued by the Czech Ministry of Agriculture on 10 June 2009.

### Chromosome preparations

Metaphases were prepared according to [57] with slight modifications. Briefly, the fish were injected with 0.1% colchicine solution (w/v, SIGMA), 1 ml/100 g body weight, for 45 minutes then sacrificed by overdose of anaesthetic 0.5% Phenoxyethanol (v/v, SIGMA). Kidneys were removed, dissected in 0.075 M KCl and the cell suspension free of tissue fragments was hypotonized for 8 min in 0.075 M KCl, fixed in methanol: acetic acid 3:1 (v/v) fixative, washed twice in fixative, and finally spread onto slides (Superfrost quality). Mitotic activity was not stimulated because these fish showed extremely high sensitivity to agents increasing mitotic rate. Simultaneously, the blood (around 0.5 ml) was collected from all analysed individuals by fine heparinized syringe for leukocyte culture according to the protocol of [58]. Briefly, partly washed leukocytes were cultivated in 5 ml of a complete medium composed of TC 199 (SIGMA, St. Louis, MO, USA), 10% FBS Superior (Biochrom, Berlin, Germany), 0.5% Antibiotic Antimycotic Solution (SIGMA), 1% Kanamycin monosulfate (SIGMA), 1% LPS (SIGMA), 0.2% PHA H15 (Remel, Lenexa, KS, USA) and 0.175ųl Mercaptoethanol (SIGMA) at 19.5°C for 6–7 days, then 2 drops of the 0.1% colchicine were added for 45 minutes at RT and cells harvested as for the direct preparation described above.

### Fluorescence *in situ* hybridization (FISH) and comparative genomic hybridization (CGH)

Probes for *in situ* hybridization experiments were produced either by PCR (FISH probes) or directly from the genomic DNA (CGH probes). Probes were indirectly labelled with haptens (biotin and digoxigenin) by means of nick translation (whole genomic DNA and FISH probe longer than 600 bp) using the Roche Nick Translation Mix (Roche, Mannheim, Germany; Cat.No. 11745808910) according to the manufacturer’s instructions. Shorter DNA fragments were labelled by PCR using the Roche PCR DIG Labeling Mix (Cat.No. 11585550910). The biotin-dUTP labelled probes (Roche, Cat. No. 11093070910) were detected by either the Invitrogen Cy™3-Streptavidin (Invitrogen, San Diego, CA, USA; Cat.No. 43–4315) or by the FITC-Streptavidin (Cat.No. 43–4311). The digoxigenin-dUTP labelled probes (Roche, Cat.No. 11093088910) were detected by either the Roche Anti-Digoxogenin-Fluorescein (Cat.No. 11207741910) or by the Anti-Digoxigenin-Rhodamin (Cat.No. 11207750910). An unlabelled DNA competitor for suppression of nonspecific hybridization of fragment size of 100–200 bp was added with 20-fold the concentration of the DNA probe in CGH experiments. The CGH DNA probe concentration was 1 ug per reaction for both genomes compared. An aging of chromosome preparations at 37°C for 3 hours was carried out prior to each of the hybridization experiment. Pepsinization, hybridization and detection were carried out under conditions as described by [59].

All rDNA FISH probes were constructed using published, mostly generally used PCR primer sets of the 45S rDNA unit to cover its major regions and to map them physically onto chromosomes.

### PCR amplification of FISH probes and the analysis of the 45S rDNA unit

All primer sets used in this study are summarized in Table 1. Primers nesting within the 45S rDNA unit relevant for this study are shown in Figure 1. Thermal profiles were used according to references given in Table 1. FISH probes were constructed from PCR conducted on the respective species as they were later hybridized. All sequences used in this study as FISH probe or in the molecular-biological analyses of the 45S rDNA unit were deposited in the GenBank [60] under accession numbers JQ731749 - JQ731760.

**Table 1:** PCR primers used in this study

**Name**	**Region of DNA/FISH probe**	**Primer sequence (5′to3′)**	**Ref.**
28S A	3′ end of the 28S rDNA involving the regions A and B, F primer	AAA CTC TGG TGG AGG TCC GT	[61]
28S B	Internally nested in the regions A and B of the 28S rDNA, R primer	CTT ACC AAA AGT GGC CCA CTA	[61]
28S C1	5′ end of the 28S rDNA adjacent to the ITS2, F primer	ACC CGC TGA ATT TAA GCA T	[62]
28S D2	Internally nested in the region C3 involving D2, C2, D1, C1, R primer	TCC GTG TTT CAA GAC GGG	[63]
ITS1	3′ end of the 18S rDNA adjacent to the ITS1, F primer	TCC GTA GGT GAA CCT GCG G	[64]
ITS2	3′ end of the 5.8S rDNA adjacent to the ITS2, R primer	GCT GCG TTC TTC ATC GAT GC	[64]
ITS3	5′ end of the 5.8S rDNA adjacent to the ITS1, F primer	GCA TCG ATG AAG AAC GCA GC	[64]
ITS4	5′ end of the 28S rDNA adjacent to the ITS2, R primer	TCC TCC GCT TAT TGA TAT GC	[64]
NS1	5′ end of the 18S rDNA F primer	GTA GTC ATA TGC TTG TCT	[64]
NS2	18 S rDNA R primer	GGC TGC TGG CAC CAG ACT TGC	[64]
NS3	18 S rDNA F primer	GCA AGT CTG GTG CCA GCA GCC	[64]
NS4	18 S rDNA R primer	CTT CCG TCA ATT CCT TTA AG	[64]
NS5	18 S rDNA F primer	AAC TTA AAG GAA TTG ACG GAA G	[64]
NS6	18 S rDNA R primer	GCA TCA CAG ACC TGT TAT TGC CTC	[64]
NS7	18 S rDNA F primer	GAG GCA ATA ACA GGT CTG TGA TGC	[64]
NS8	3′ end of the 18S rDNA R primer	TCC GCA GGT TCA CCT ACG GA	[64]
RTX1F1	Rex1 F primer	TTC TCC AGT GCC TTC AAC ACC	[28]
RTX1R3	Rex1 R primer	TCC CTC AGC AGA AAG AGT CTG CTC	[28]
RTX3F1	Rex3 F primer	TAC GGA GAA AAC CCA TTT CG	[65]
RTX3F2	Rex3 F primer	AAC ACC TTG GCT GCG CCT AG	[65]
RTX3F3	Rex3 F primer	CGG TGA YAA AGG GCA GCC CTG	[28]
RTX3R1	Rex3 R primer	AAA GTT CCT CGG TGG CAA GG	[65]
RTX3R2	Rex3 R primer	CCR GGG GTG GAT GAR RTC CGC CC	[65]
RTX3R3	Rex3 R primer	TGG CAG ACN GGG GTG GTG GT	[28]
RTX6F	Rex6 F primer	TAA AGC ATA CAT GGA GCG CCA C	[28]
RTX6R	Rex6 R primer	GGT CCT CTA CCA GAG GCC TGG G	[28]

### Cloning, sequencing and sequences analysis

PCR products were cloned using the QIAGEN PCR Cloning Kit and QIAGEN EZ Competent Cells (Qiagen, Hilden, Germany); the plasmids were isolated from the cells with Qia PREP Spin Miniprep Kit according to the manufacturer’s instructions. The primary PCR products were first sequenced on the ABI 3130 Genetic Analyzer (Applied Biosystems, Hitachi, Foster City, CA, USA) using the BigDye Terminator Cycle Sequencing Kit (Applied Biosystems). Furthermore, cloned DNA fragments that were later applied as FISH probes were commercially sequenced by Macrogen (Seoul, South Korea). The commercially obtained sequences were subjected to online megablast or discontiguous megablast [66] searches at the National Center for Biotechnology Information (NCBI) [67], where their similarity to the sequences deposited in the GenBank databases was checked.

### Microscopy and image processing

Chromosome preparations were analysed with the Provis AX70 Olympus microscope equipped with standard fluorescence filter sets. Gray-scale hybridization signals on chromosomes and/or DAPI counterstained chromosomes were captured by the CCD camera (DP30W Olympus). Using the Olympus Acquisition Software, black and white images were pseudo-coloured and superimposed with the software MicroImage. The colour images have been analyzed and processed with Adobe Photoshop, Version CS5. The chromosomes were classified using the nomenclature proposed by [68]. Karyotypes based on the Giemsa-stained chromosomes were produced using the IKAROS (Metasystems) software. Chromosomal formulas were formed according to [69].

## Abbreviations

AFLP: Amplified fragment length polymorphism;CGH: Comparative genomic hybridization;CMA3: Chromomycin A_3_;DAPI: 4′, 6-diamidino-2-phenylindole;DAPI/CMA3+/−: DAPI/CMA_3_ positive/negative signals;FISH: Fluorescence *in situ* hybridization;IAPG: Institute of animal physiology and genetics;kyrs BP: Thousand years before presence;NF: “Nombre fundamental” chromosome arm number;NGS: Next generation sequencing;NOR: Nucleolar organizer DNA;rDNA: Ribosomal DNA;TE: Transposable elements;2n: Diploid chromosome number

## Competing interests

There are no competing interests to declare.

## Authors’ contributions

RS designed and performed experiments FISH and CGH and co-drafted the manuscript, ZM and AS performed FISH experiments, ZM performed PCRs and sequenced PCR products and co-drafted the manuscript, GS and JF collected and raised material, JB and RS prepared chromosome preparations, MR partly contributed to digital processing of images, PR designed the study and co-drafted the manuscript. All authors read and approved the final manuscript.
